# Introduction to ‘Development of bio-orthogonal tools’

**DOI:** 10.1039/d2cb90045a

**Published:** 2022-12-09

**Authors:** Yan Zhang, Chengqi Yi

**Affiliations:** a Nanjing University China; b Peking University China

## Abstract

Yan Zhang and Chengqi Yi introduce the *RSC Chemical Biology* themed collection on ‘Development of bio-orthogonal tools’.
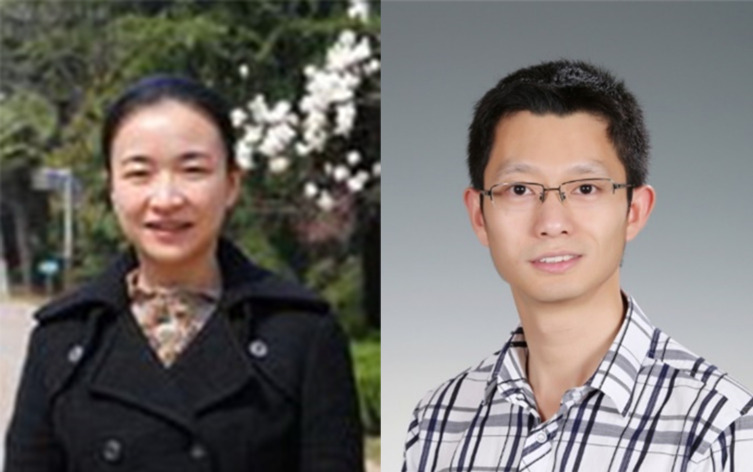

This themed collection includes four articles that describe the development of bioorthogonal molecular tools. The establishment of bioorthogonal chemistry was a milestone in chemical biological research. The pioneer in the field of bioorthogonal chemistry, Carolyn R. Bertozzi, was one of the Nobel laureates in chemistry in 2022. Significant progress has been made in recent years using bioorthogonal tools to elucidate complex biological systems. This collection assembles new contributions that showcase the tremendous promise of bioorthogonal tools.

As part of this collection, Xiang Zhou and his colleagues reviewed the latest research reports on the application of labeling and sequencing DNA and RNA modifications *via* bio-orthogonal reactions (https://doi.org/10.1039/d2cb00087c). Due to the low toxicity, high specificity, and convenience of bio-orthogonal reactions, they are widely used in the labeling and detection of intracellular DNA and RNA molecules as well as their modifications.

The contribution from the Kasteren lab synthesized a close mimic of phenylalanine called methyltetrazinylalanine (MeTz-Ala), which was then incorporated into a peptidyl inhibitor (https://doi.org/10.1039/d2cb00120a). It was confirmed that the resultant tetrazine-containing probe has a similar cathepsin inhibition pattern to the parent inhibitor, and enables visualization of the probe–protease conjugates in live cells by inverse electron demand Diels–Alder (IEDDA) ligation with transcyclooctene (TCO)-modified fluorophores. This work proves that tetrazines can be used as live-cell-compatible, minimal bioorthogonal tags in activity-based protein profiling.

Wang's group summarized the structural design rationales and bioorthogonal features of stem peptide-mimicking probes (https://doi.org/10.1039/d2cb00086e) that target bacterial peptidoglycan (PGN) and help to reveal specific steps in PGN biosynthesis. They highlighted the use of d-amino acids modified with bioorthogonal functionalities in labeling various microbial cells to present models for PGN biosynthesis. Fluorescent dyes are frequently linked with bioorthogonal groups for *in situ* labeling of biomacromolecules incorporating corresponding bioorthogonal functionalities.

Finally, the contribution from Greineder and co-workers (https://doi.org/10.1039/d2cb00030j) describes a library of water-soluble pH-sensitive and photostable fluorescent dyes with a common framework of rhodamine 6G.

Taken together, the papers collected in this themed collection represent the state of the art in bio-orthogonal tools. More research methods capable of carrying out bio-orthogonal reactions should be developed, which will lead to more opportunities for the area. We hope that our readers will find these contributions equally inspiring and thought-provoking.

## Supplementary Material

